# Disruption of Type I Interferon Induction by HIV Infection of T Cells

**DOI:** 10.1371/journal.pone.0137951

**Published:** 2015-09-16

**Authors:** David Jesse Sanchez, Daniel Miranda, Matthew D. Marsden, Thomas Michael A. Dizon, Johnny R. Bontemps, Sergio J. Davila, Lara E. Del Mundo, Thai Ha, Ashkon Senaati, Jerome A. Zack, Genhong Cheng

**Affiliations:** 1 Pharmaceutical Sciences Department, College of Pharmacy, Western University of Health Sciences, Pomona, California, United States of America; 2 Department of Microbiology, Immunology and Molecular Genetics, University of California Los Angeles, Los Angeles, California, United States of America; 3 UCLA AIDS Institute, David Geffen School of Medicine at UCLA, Los Angeles, California, United States of America; 4 Department of Medicine, Division of Hematology and Oncology, David Geffen School of Medicine at UCLA, Los Angeles, California, United States of America; University of Regensburg, GERMANY

## Abstract

Our main objective of this study was to determine how Human Immunodeficiency Virus (HIV) avoids induction of the antiviral Type I Interferon (IFN) system. To limit viral infection, the innate immune system produces important antiviral cytokines such as the IFN. IFN set up a critical roadblock to virus infection by limiting further replication of a virus. Usually, IFN production is induced by the recognition of viral nucleic acids by innate immune receptors and subsequent downstream signaling. However, the importance of IFN in the defense against viruses has lead most pathogenic viruses to evolve strategies to inhibit host IFN induction or responses allowing for increased pathogenicity and persistence of the virus. While the adaptive immune responses to HIV infection have been extensively studied, less is known about the balance between induction and inhibition of innate immune defenses, including the antiviral IFN response, by HIV infection. Here we show that HIV infection of T cells does not induce significant IFN production even IFN I Interferon production. To explain this paradox, we screened HIV proteins and found that two HIV encoded proteins, Vpu and Nef, strongly antagonize IFN induction, with expression of these proteins leading to loss of expression of the innate immune viral RNA sensing adaptor protein, IPS-1 (IFN-β promoter stimulator-1). We hypothesize that with lower levels of IPS-1 present, infected cells are defective in mounting antiviral responses allowing HIV to replicate without the normal antiviral actions of the host IFN response. Using cell lines as well as primary human derived cells, we show that HIV targeting of IPS-1 is key to limiting IFN induction. These findings describe how HIV infection modulates IFN induction providing insight into the mechanisms by which HIV establishes infection and persistence in a host.

## Introduction

Global infection by Human Immunodeficiency Virus (HIV), the cause of Acquired Immune Deficiency Syndrome or AIDS, has reached a pandemic scale. This global burden of HIV infection and the lack of an efficacious HIV vaccine underscore the need for a deeper understanding of the host-pathogen interactions involved in HIV infection, especially the immune response to HIV infection [1, 2]. As a member of the retrovirus family, HIV not only can effectively establish chronic infection of CD4+ T cells and macrophages by integrating its genome into host cell DNA, but HIV also remains persistently in long-lived CD4+ T cells through establishment of a latent, reversibly non-expressing, provirus. Long-term depletion of infected and bystander CD4+ T cells manifests as the profound immunodeficiency of AIDS. A number of mechanisms have been proposed to describe the reduction of CD4+ T cells over time involving both direct and indirect pathogenic mechanisms that are cytotoxic to CD4+ T cells [3–7]. Additionally, years of unabated HIV infection leads to degradation of lymph node architecture that would nurture the development of mature, functional CD4+ T cells, further lowering the ability for the CD4+ T cell population to fully recover even after the effective management of HIV infection by administration of current therapies [8].

While the mechanisms by which long term HIV infection leads to a compromised adaptive immune response is increasingly well characterized, much less is known of how HIV modulates the innate immune response. After any virus is introduced into a human host, receptors of the innate immune system will bind to pathogen associated molecular patterns (PAMPs) present as structural components or replication intermediates of the virus [9, 10]. Commons PAMPs of viruses include exotic nucleic acids as well as certain proteins, each of which is bound by a type of pattern recognition receptor (PRR) of the innate immune system that subsequently triggers an intracellular signalling cascade. These signals lead to the induction of transcription of innate immune cytokines and other antiviral proteins. As primary infection with HIV progresses to chronic infection, innate recognition of the constant signs of viral infection may be a primary cause that leads to a perturbation of the overall homeostasis of the infected individual as the innate immune system begins to persistently respond by the release of cytokines and effector molecules [11, 12]. Each of these responses are not in and of themselves detrimental to the infected individual. However, just as a proper immune response is a concerted orchestra of responses, chronic HIV infection leads to a disharmonious set of responses. As the overall pathophysiology of HIV infection and AIDS has been studied more completely, understanding the perturbation of physiology by the cacophony of cytokines has given insight into how we should target the innate immune responses to allow for proper clearance of HIV.

In particular, the quintessential innate immune cytokines for limiting the replication of viruses, and thus key in eliminating all types of viruses, are Type I Interferons (IFNs) [13, 14]. Recognition of viral components by an innate immune receptor, most notably members of the Toll-like receptors (TLR) and RIG-I like RNA helicases (RLR) families of receptors, will lead to signal transduction that induces the transcriptional upregulation and secretion of IFN [9, 10]. Released IFNs will signal in autocrine and paracrine pathways via binding to the Interferon-α/β receptor (IFNAR), leading to induction of JAK/STAT signalling. Signalling through IFNAR leads to the transcriptional upregulation of a veritable pantheon of effector genes, the Interferon Stimulated Genes (ISGs) including modulators of cellular physiology, activators of the immune system as well as directly antiviral proteins [15, 16]. Though some groups have shown specific anti-ISG mechanisms mediated by individual HIV proteins [17–21], there is currently little evidence describing why one of the other hundreds of ISGs does not completely block HIV infection.

Our goal was to understand why HIV is not blocked by the huge array of ISGs and system of IFN that should stop progression of infection. To understand if our host innate immune system is able to detect HIV infection and generate proper innate immune responses, we looked at induction of IFN in cells infected with HIV. Here we show how IFN infection by infected CD4+ T cells is directly modulated by HIV infection.

## Materials and Methods

### Virus Infection

Infectious HIV-1 (NL4.3) was produced by transient transfection of 293FT [22, 23] cells using Lipofectamine2000 reagent (Invitrogen). CEM T cells (2×10^6^) were infected with 400 ng of p24 protein by spinoculation in a flat-bottomed 24-well plate in 400 μl volume of media (RPMI 1640 media + 10% fetal bovine serum) containing virus and 10 μg/ml polybrene. The plate was centrifuged at 1200xg for 2 hr at 25°C. Then cells were washed and incubated in 5 ml of media for 48 hours. The following reagent was obtained through the NIH AIDS Reagent Program, Division of AIDS, NIAID, NIH: CEM CD4+ Cells from Dr. J.P. Jacobs [24]

For primary cell infections, peripheral blood mononuclear cells were purified from healthy human blood by Ficoll-Paque PLUS (GE healthcare) separation. Primary CD4+ T cells were then isolated by negative selection using the CD4+ T Cell Isolation Kit II (Miltenyi Biotec) and an AutoMACS cell separator according to the manufacturer’s instructions. Cells were costimulated for 2 days prior to infection by culturing in RPMI Medium 1640 (Invitrogen) supplemented with 10% FBS, Pen/Strep, and 20 units/ml of interleukin-2 (Roche) in the presence of soluble anti-CD28 and plate-bound anti-CD3 antibodies as previously described [25]. Infections were done as above with 50 ng of HIV p24 used to infect 10^6^ cells.

During harvesting, cells were pelleted by centrifugation then 100 μl of supernatant was removed and added to 900 μl of 2% Triton-X-100. Supernatants were stored at 4°C then assayed by ELISA for IFN (PBL VeriKine Interferon Detection Kits) or p24 levels (Beckman Coulter). For flow cytometry, cell pellets were fixed in 2% paraformaldehyde, permeabilized using 0.02% Tween 20, and then stained for intracellular p24 protein detection using KC57-Phycoerythrin (Beckman Coulter). Cell pellets were frozen at -80°C before use in western analysis.

For Vesicular Stomatitis Virus (VSV, Indian Serotype, a gift of Dr. Glen Barber) was used at an MOI of 0.1 to infect CEM cells. Aliquots of cells were monitored for GFP expression by flow cytometry and supernatants were assayed for IFN production by ELISA.

### Ethics Statement

This study did not include human subjects. Peripheral blood mononuclear cells were obtained through the UCLA Blood Bank in an anonymous fashion. Cells from anonymous sources are not considered human subjects, and are thus not subject to Institutional Review Board review.

### Lysate and RNA Preparation

Purified HIV virion lysate was purchased from Advanced Biotechnologies Inc (Cat #10-119-100). Biochemical treatments were done with RNaseA, RNaseV1, and Alkaline Conditions according to manufactures protocol (Ambion, Applied Biosystems). After treatment the nucleic acid components were purified by standard phenol:chloroform extraction. Transfections of the lysates are as described below. Cells were transfected with 1 ng of lysate per well of a 24 well dish. Experiments were repeated on three separate occasions and representative data is shown.

### Reporter Assays and Transfections

HEK 293T cells or murine embryonic fibroblasts (MEFs) were transfected with IFN-β luciferase and Renilla luciferase plasmids at a 100 ng:50 ng ratio for a set of triplicates in three wells of a 24 well dish using Fugene 6 (Roche). RNA or lysates were transfected using Lipofectamine 2000 (Invitrogen) according to manufacturers instructions. All transfections done solely with DNA were done with Fugene 6 (Roche) according to the manufacturers instructions. ISRE and κB luciferase were used at a 200 ng:50 ng ratio for a set of triplicates in three wells of a 24 well dish. Inducers (e.g. IPS-1) and blockers (e.g. Vpu or Nef) were used at 200 ng:1000ng ratio for a set of triplicates in three wells of a 24 well dish. Transfections were allowed to proceed for 18 to 24 hours, harvested and analyzed using a Dual-Luciferase Reporter Assay (Promega) according to manufacturer’s protocol. Infections with Vesicular Stomatitis Virus (VSV) were done 12 hours after transfection for 18 to 24 hours using an MOI of 0.01. Afterwards cells were fixed and analysed by flow cytometry for levels of GFP. Experiments were repeated on at least three separate occasions and representative data is shown.

### Protein Stability Assays

HEK 293T cells were transfected as above but were lysed in SDS-Loading buffer for whole cell lysates. Infected CEM T cells were treated in a similar fashion. Cell lysates were separated and western blotted for the protein as well as tubulin. Signal for each protein was detected by digital capture of HRP catalyzed light release using a BioRad ChemiDoc XRS System with Quantity One Software. The individual band intensities were quantitated and each target protein was normalized to the level of tubulin signal present. Experiments were repeated on five separate occasions for IPS-1 and three occasions for other molecules being assayed.

### Mutant Virus Engineering

The NLΔvpuΔnef clone (referred to as Delta Vpu/Nef in figures) was produced by partially digesting the denv(Wt) plasmid with *Xho*I to cleave only the site within the nef open reading frame [26]. An inactivating frameshift mutation was then introduced by performing an end-filling reaction using T4 DNA polymerase followed by re-ligation of the blunted ends. The *Sal*I-*Bam*HI region of this vector was then replaced with the corresponding region of a previously described 3' HIV clone that is deficient in vpu and nef [27], to produce a replication competent NL4-3-based viral genome defective in only Vpu and Nef expression.

### Note on Values Graphed in Figures

In an effort to show genuine quantitative data, we do not show fold induction for any experiment but instead show the actual values obtained in an experiment normalized to the internal standard. We feel this gives a more genuine representation of data and that the data is not masked through the use of fold induction values, especially in reporter assays. Consequently, the values in different experiments may vary but the interpretation remains the same and is more consistent with the biology of each individual experiment.

## Results

### HIV Infection Could but Does Not Induce IFN

**Fig 1:**
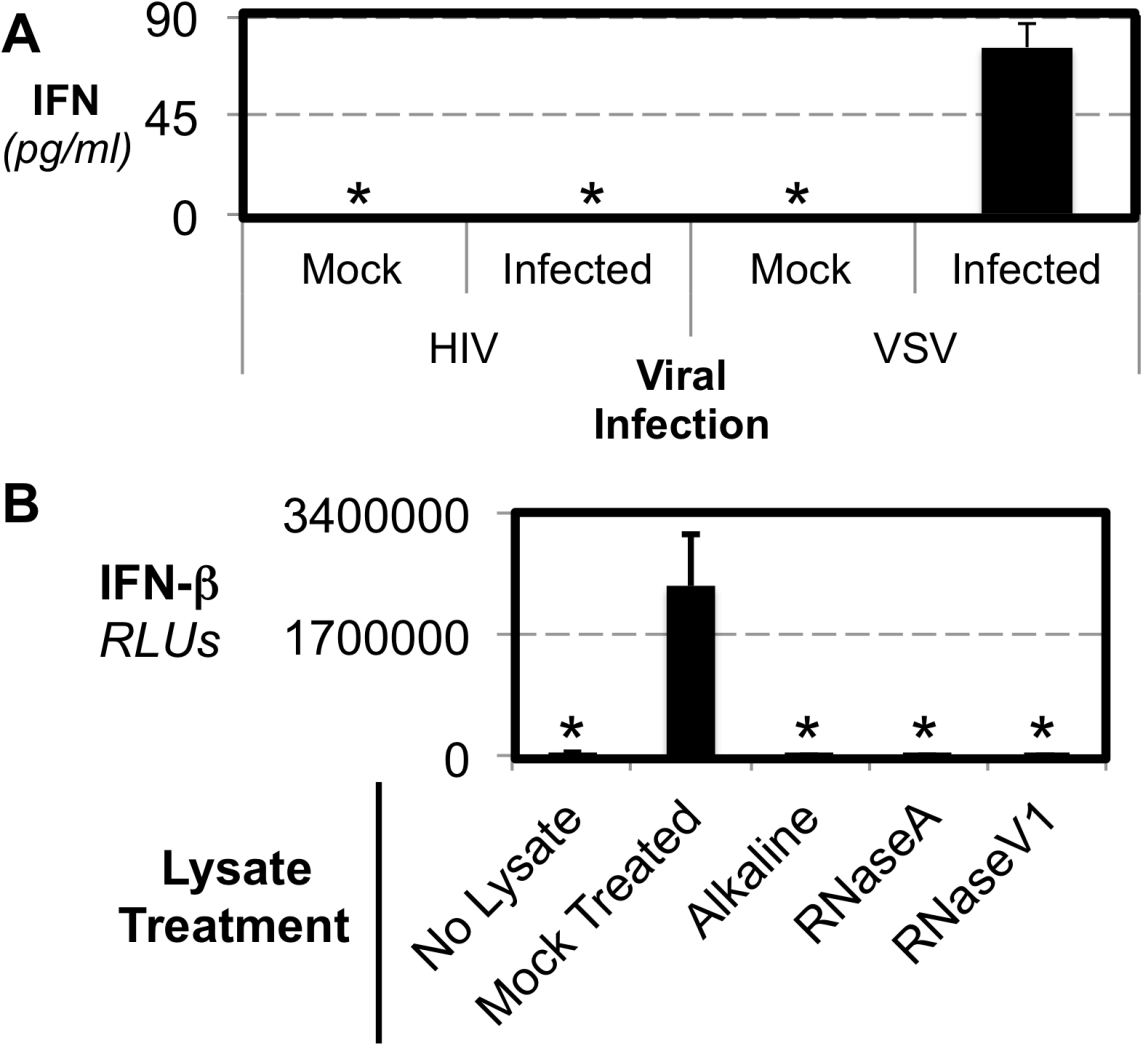
HIV Infection Could But Does Not Induce IFN. (A) CEM T cells were infected with either HIV or VSV and after 48 hours the levels of IFN-α in supernatants were determined by ELISA. (B) Lysates from purified HIV virions were transfected along with an IFN-β reporter into HEK 293T cells. Lysates were treated as indicated. IFN-β reporter activation was measured, and graphed in relative light units normalized to cotransfected Renilla luciferase (designated RLUs).

To begin understanding how HIV modulates global ISG function, we started with a classical measurement of IFN-α release after HIV infection of CD4+ T cells. We used the CD4+ T cell line CEM that responds with IFN-α release upon Vesicular Stomatitis Virus (VSV) infection as monitored by ELISA (Fig 1A). We were surprised to observe that HIV infection of these cells does not lead to any appreciable IFN release (Fig 1A).

To determine if the necessary PAMPs were present in HIV to induce IFN via the innate immune system we used lysates from purified HIV virions that were transfected into cells with reporters for IFN-β promoter activity. Transfection of HIV virion lysates strongly induces IFN-β promoter activity (Fig 1B) confirming the presence of at least one IFN inducing PAMP in HIV virions. The ability of HIV lysates to induce IFN is via virion associated RNA as addition of RNAse ablates the capacity of the lysates to induce IFN (Fig 1B). Thus, though infection by HIV does not induce IFN, virion associated RNA is a PAMP that has the potential to induce IFN. Intracellular viral RNA ligands are recognized by families of cytoplasmic RLR proteins. RLRs recognize RNA and induce IFN through the use of the signalling adaptor protein IPS-1 (also called CARDIF, MAVS or VISA) to transduce downstream signalling [28–31].

### HIV Proteins Block the Antiviral Signalling of IPS-1

**Fig 2:**
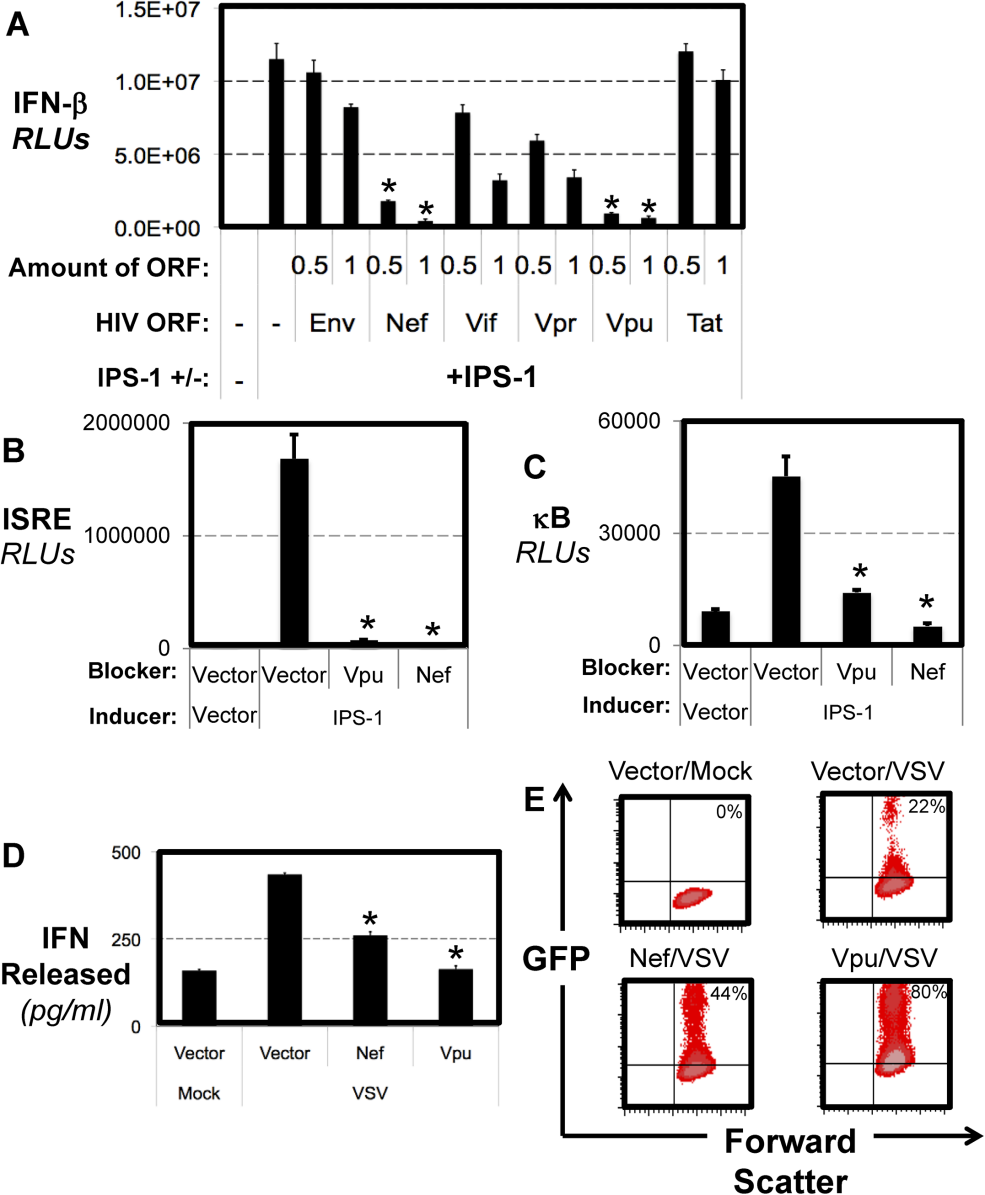
HIV Proteins Block the Antiviral Signalling of IPS-1. (A) IPS-1 overexpression was used to induce IFN-β reporters. HEK 293T cells were cotransfected with expression plasmids for different HIV protein products and the levels of IFN-β luciferase were determined. Reporter activation in A-C was measured, and graphed in relative light units normalized to cotransfected Renilla luciferase (designated RLUs). * are values with p<1x10^-4^ as compared to IPS-1 alone. (B) HEK 293T cells were co-transfected with IPS-1 and vector control, *Vpu* or *Nef* and an ISRE reporter. * are values with p<1x10^-3^ as compared to IPS-1 alone. (C) HEK 293T cells were co-transfected with IPS-1 and vector control, *Vpu* or *Nef* and a κB reporter. * are values with p<1x10^-3^ as compared to IPS-1 alone. (D) CEM cells were stably transduced with either Vpu or Nef. The cells were then infected with VSV that expresses GFP upon active infection. After 48 hours, supernatants were collected and monitored by ELISA for the amount of secreted IFN. * are values with p<1x10^-4^ as compared to vector infected cells. (E) In addition, cells were collected from the infection in D and assayed for GFP levels, an indicator of VSV replication in the culture.

Recent studies have identified many viral proteins such as NS3/4A of HCV and NS1 of influenza virus that can block IFN induction through the IPS-1pathway allowing the virus to replicate unimpeded by IFN [29, 32–35]. Since we did not see induction of IFN by HIV in Fig 1A, we suspected that HIV encodes a mechanism to block IFN induction. To determine the encoded product of HIV that antagonizes IPS-1signalling, we expressed different HIV proteins in the presences of overexpressed IPS-1. Overexpression of IPS-1is sufficient to induce IFN. Upon screening HIV viral ORFs for their ability to block IPS-1mediated IFN induction, we observed that expression of either the *Vpu* or *Nef* ORF strongly inhibits IFN induction, while other ORFs such as *Env* and *Tat* did not have significant effects (Fig 2A). The gene products of *Vif* and *Vpr* are known to affect IRF-3 mediated IFN induction and thus we were not surprised to see a modest effect induced by high levels of these proteins because part of IPS-1signalling goes through IRF-3 [36].

RLR-mediated signal transduction via IPS-1diverges to multiple downstream pathways leading to the independent activation of transcription factors such as IRF-3/7 and NF-κB, which bind to the ISRE and κB sites of IFN promoter elements respectively to promote its transcription. Transfection of cells with *Vpu* or *Nef* led to a block of IPS-1 induced activation of both ISRE and NF-κB reporters (Fig 2B and 2C). These results support the idea that the effects of Vpu and Nef on the IPS-1-mediated IFN Signalling pathway occur prior to the divergence into the specific IRF-3/7 and NF-κB axes.

To further address the functional relevance of these protein products to block the endogenous RLR/IPS-1 dependent pathway, we generated CEM cell lines that were infected with retroviruses to stabely express either *Vpu* or *Nef*. We examined the ability of Vpu and Nef to inhibit IFN production and the anti-viral state induced by infection with VSV using cells stably expressing Vpu or Nef. As shown in Fig 2D, IFN induction after VSV infection of these CD4+ T cells is reduced in cells that have stable expression of either Vpu or Nef but not mock transduced cells. To observe the consequence of such abrogated IFN induction, we monitored GFP production as the strain of VSV we used encodes GFP. We saw that VSV replicates at an increased rate in Vpu and Nef expressing cells as compared to control cells showing the ability of these two HIV proteins to block establishment of an effective antiviral state (Fig 2E). Interestingly, the trend of *Vpu* expression leading to less IFN release is reflected in the relative increase in VSV replication as monitored by GFP expression. Overall, this shows that both Vpu and Nef can block IPS-1 signalling and consequently block induction of IFN release and inhibit establishment of an efficacious antiviral state.

### Vpu and Nef Lead to Diminished IPS-1 Expression

**Fig 3:**
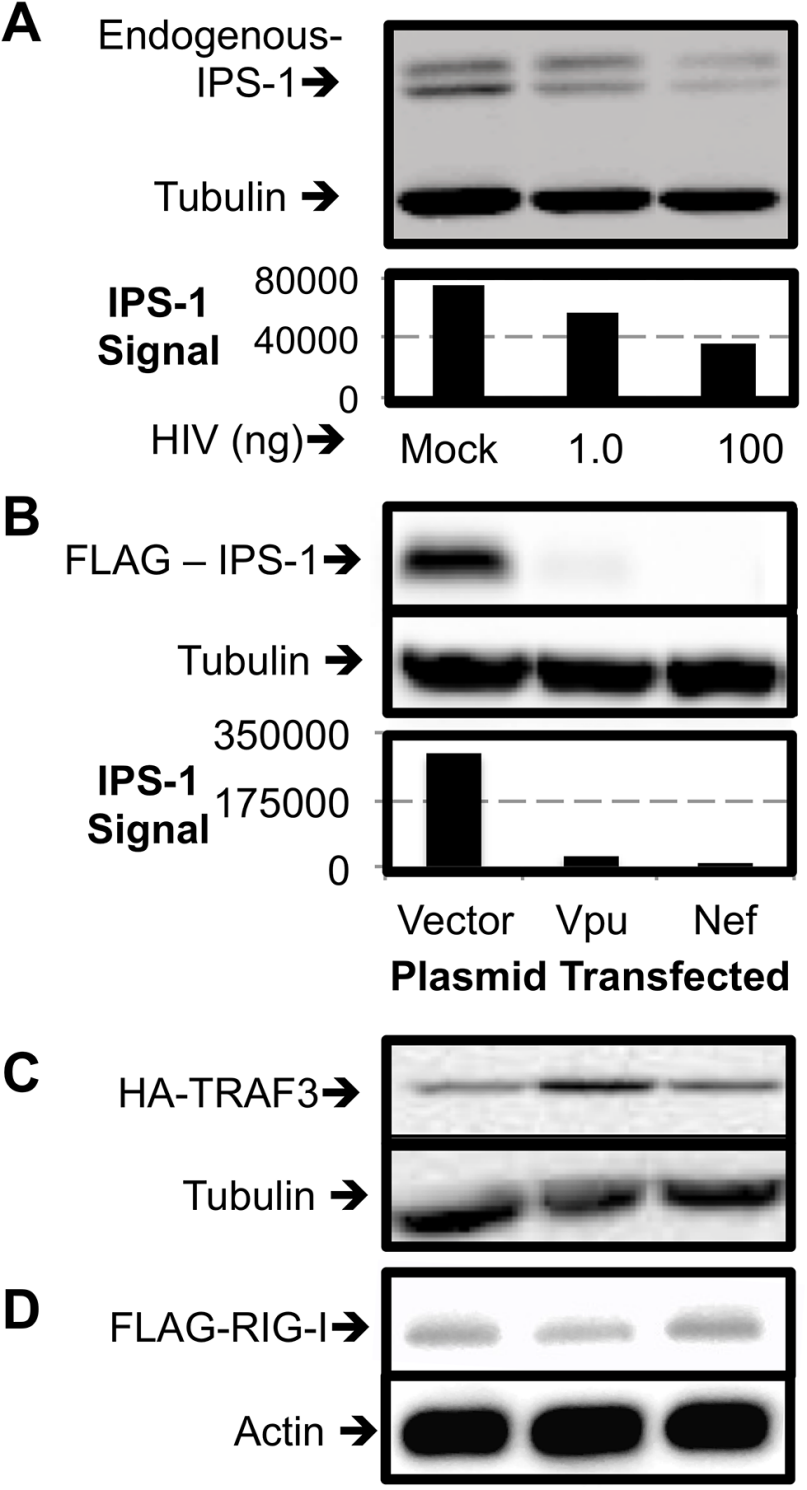
Vpu and Nef Lead to Diminished IPS-1 Expression. (A) CEM T cells were infected with HIV and after 24 hours cell lysates were prepared and blotted for levels of endogenous IPS-1 and tubulin. In A and B HRP catalysed light release was digitally recorded and displayed as a picture as well as quantified as a measure of the target band intensity divided by the intensity of the tubulin cell loading control. (B) HEK 293T cells were transfected with IPS-1 expression plasmid with a FLAG tag and the indicated plasmids. Whole cell lysates were analysed by Western blot for the expression of FLAG and tubulin. (C) HEK 293T cells were transfected with TRAF3 expression plasmid with a HA tag and the indicated plasmids. Whole cell lysates were Western blotted for the expression of HA and tubulin. (D) HEK 293T cells were transfected with RIG-I expression plasmid with a FLAG tag and the indicated plasmids. Whole cell lysates were Western blotted for the expression of FLAG and actin.

Vpu and Nef are well known to lead to degradation of host molecules in HIV infected cells, such as the CD4 molecule [37]. These previous studies guided us to investigate whether Vpu or Nef block IPS-1function by inducing IPS-1degradation or instability. In the context of HIV infection of CEM T cells, we determined that endogenous IPS-1 was degraded after HIV infection (Fig 3A). In addition, we observed that Vpu and Nef expression leads to complete ablation of co-transfected IPS-1 protein levels (Fig 3B).

In both experiments, endogenous tubulin was not affected so we were confident that this effect was not due to cytotoxic effects of the proteins. To further show that this was not nonspecific degradation or a block to translation, we monitored TRAF3. TRAF3 is a protein that coordinates IPS-1 signalling by direct interaction with activated IPS-1 and serves almost as a molecular bridge to downstream proteins [38, 39]. Using a setup that parallels Fig 3B, we determined that neither Vpu nor Nef co-transfection leads to degradation of TRAF3 (Fig 3C). Additionally, expression of RIG-I, an RLR upstream of IPS-1, is still seen when co-transfected with either Vpu or Nef (Fig 3D). Thus HIV infection leads to specific IPS-1 degradation and either Vpu or Nef is sufficient to induce degradation of IPS-1, which would inhibit IFN induction.

### Vpu and Nef Do Not Block Other IFN-Inducing Signalling

**Fig 4:**
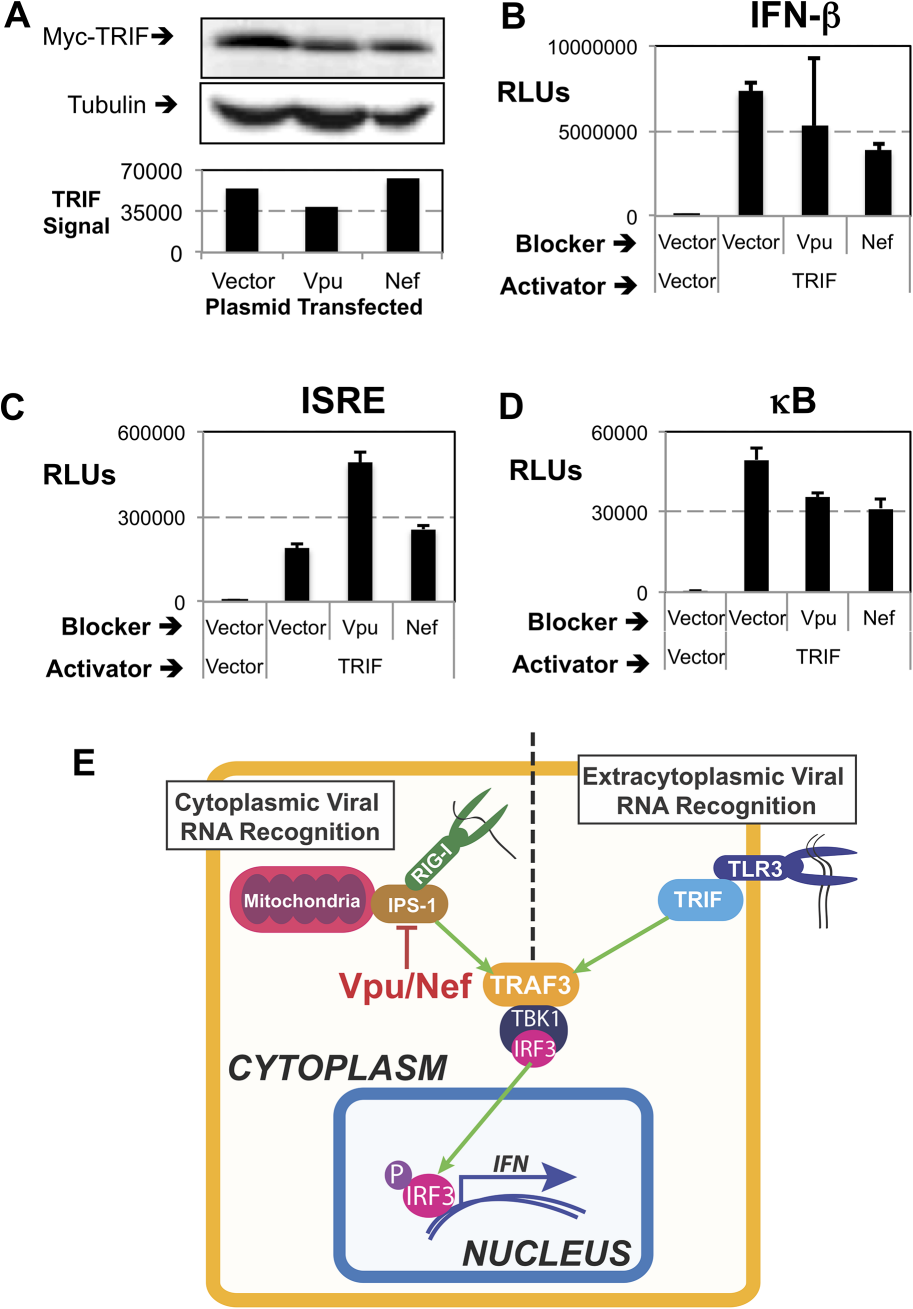
Vpu and Nef Do Not Block Other IFN-Inducing Signaling. (A) Cells were transfected with TRIF expression plasmids containing a Myc tag fused to TRIF and the indicated plasmids. Whole cell lysates were analysed by Western blot for the levels of Myc and endogenous tubulin. HRP catalysed light release was digitally recorded and displayed as a picture as well as quantified as a measure of the target band intensity divided by the intensity of the tubulin cell loading control. (B-D) TRIF overexpression was used to induce the IFN-β (B), ISRE (C) or κB (D) reporters. For B and C, Vpu and Nef induced changes have p values >0.0001. For D, the decreased in kB signalling induced by B and C have a p value > 0.001. In addition, cells were cotransfected with expression plasmids for either *Vpu* or *Nef* and the levels of luciferase were analysed. Reporter activation was measured, and graphed in relative light units normalized to cotransfected Renilla luciferase (designated RLUs). (E) A Diagram of cellular IFN induction pathways. The left hand side contains the Cytoplasmic Viral RNA Recognition pathway including RIG-I and IPS-1. The right hand side has an extracytoplasmic viral RNA recognition pathway exemplified by TLR3/TRIF signalling. The convergence of the two pathways is focused at TRAF3 with downstream signalling leading to TBK1 mediated activation of IRF-3.

Induction of IFN can occur via signalling from several distinct pathways. Notably, parallel signal transduction pathways converge on the activation of similar sets of transcription factors such as IRFs and NF-κB that can induce IFN transcription. Again, we used an experimental setup similar to Fig 3B to see if overexpression of *Vpu* or *Nef* could induce modulation of other proteins in the IFN induction network. TRIF is an adaptor protein for TLRs 3 and 4. It is notable in that it converges on TRAF3 and uses proteins downstream of that interaction similar to the IPS-1 pathway to induce IFN. As with IPS-1, overexpression of TRIF is sufficient to induce IFN induction. We saw that expression of neither *Vpu* nor *Nef* induced a reduction in expression of the TRIF protein (Fig 4A). In addition, TRIF signalling is not substantially inhibited by either *Vpu* or *Nef* expression (Fig 4B).

We did notice a slight decrease in *IFN-β* promoter induction in Fig 4B and tested both ISRE and κB activation to see if either was modulated by *Vpu* or *Nef* expression. While there was no decrease in ISRE activation, showing an intact IRF activation system (Fig 4C), κB activation was dampened in the presence of *Vpu* or *Nef* expression (Fig 4D). This modulation of NF-κb by *Vpu* or *Nef* expression has been seen previously by others. Overall, we see that though *Vpu* or *Nef* expression leads to a block to IPS-1 mediated IFN induction, there is no block induced by *Vpu* or *Nef* expression on TRIF mediated IFN induction (Diagrammatically Summarized in Fig 4E). This shows that there is a specific block by Vpu or Nef on IPS-1 induce signaling and not a systematic block to the IFN induction pathway.

### Vpu and Nef Are Required for Disruption of IFN Signalling by HIV Infection

**Fig 5:**
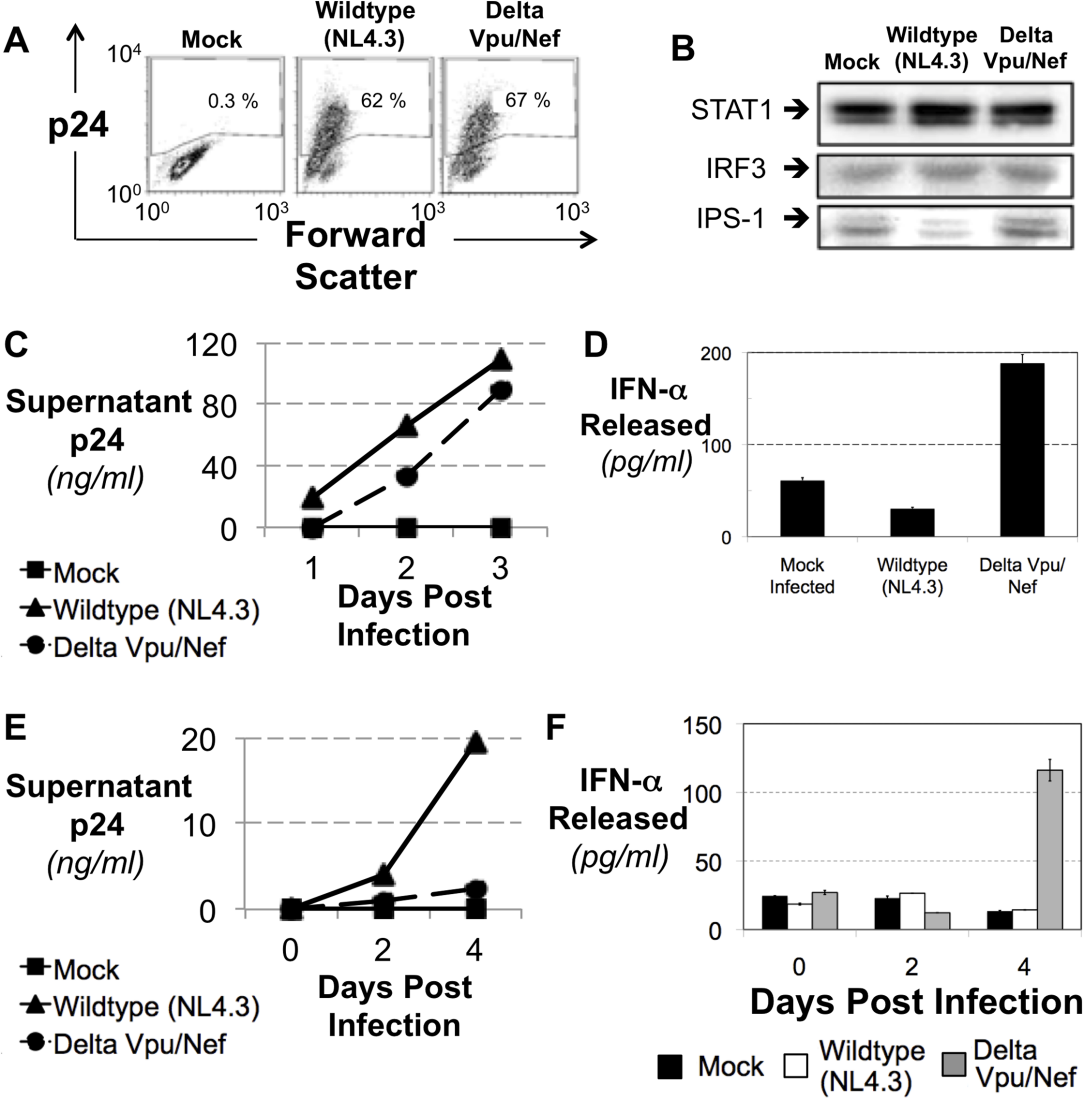
Vpu and Nef are Required for Disruption of IFN Signalling by HIV Infection. (A) A mutant of HIV was constructed lacking expression of the Vpu and Nef gene products. CEM T cells were infected with wildtype (NL4.3) or the mutant (Delta Vpu/Nef) and after 48 hours the level of intracellular p24 was analysed by flow cytometry to verify equal infection levels. (B) Protein levels of STAT1, IRF3 and IPS-1 were analysed via western blotting of cell pellets from the infection in B. (C) The level of p24 in the supernatant of infected cells was monitored by ELISA over the three days of infection. (D) The level of IFN-α was quantitated via ELISA of the supernatant of the infected cells from B. The increase in the IFN-α released from the mutant compared to the wildtype has a p value <0.00001. (E) Primary CD4+ T cells were infected with wildtype (NL4.3) or the mutant (Delta Vpu/Nef) for three days and the level of p24 in the supernatant was analysed by ELISA to monitor viral infection. (F) The level of IFN-α was quantitated via ELISA of the supernatant of the infected cells from E.

Our work thus far allows us to hypothesize that though HIV virion associated RNA has the capacity to induce IFN, degradation of IPS-1 by HIV infection will block signal transduction through this pathway. To test this, we asked how important targeting of IPS-1 by Vpu and Nef is during HIV infection. We took advantage of a mutant HIV that does not produce either the Vpu or the Nef protein upon infection (See Materials for Virus Engineering Design). We normalized infection of these viruses and analysed the cells for protein stability and IFN induction state (Fig 5A). Upon infection in CEM T cells, only wildtype HIV but not the mutant HIV lacking Nef and Vpu induced a destabilization of IPS-1 protein. Both STAT1 and IRF3 levels are constant in the early stage of either wildtype or mutant HIV infected CEM T cells (Fig 5B). Next, we revisited our initial observation that HIV infection of a T cell does not induce IFN. Using this double mutant HIV, we saw that infection by HIV that does not express Vpu or Nef now leads to IFN induction in this CEM T cell line (Fig 5C and 5D). This suggests that the Vpu- and Nef-mediated degradation of IPS-1 is at least partially involved in the disruption of IFN induction by HIV allowing HIV replication.

Finally, we went on to show that the observation of HIV modulation of IPS-1 mediated IFN induction in CEM T cells could also be seen in primary CD4+ T cells. Using primary CD4+ T cells infected with either wildtype or mutant HIV, we see that HIV that is mutant in both Vpu and Nef replicates at a rate far below wildtype HIV (Fig 5E). However, while the virus is not able to replicate as well, it leads to substantial IFN release detectable after three days of infection by the HIV mutant (Fig 5F). We attribute this induction of IFN to the lack of Vpu and Nef to counter the IPS-1 mediated induction of IFN that responds to the presence of HIV RNA in the system.

## Discussion

**Fig 6:**
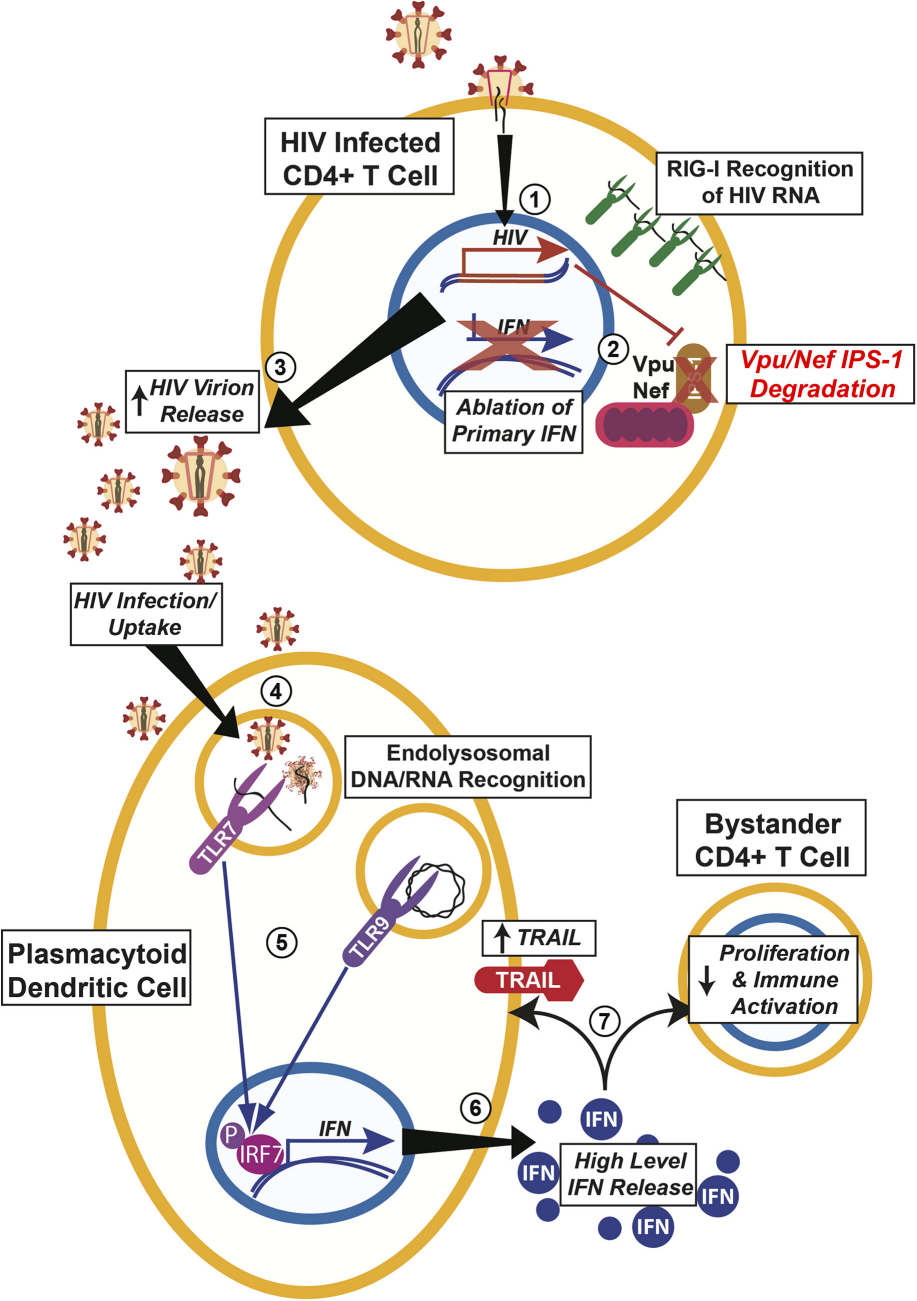
Modulation of Type I Interferon Affects Different Aspects of HIV Pathogenesis. 1. HIV infection of a target CD4+ T cell allows for proviral integration. 2. Normally HIV RNA is recognized by RIG-I and should signal through IPS-1. However, as we show here, Vpu and Nef lead to the degradation of IPS-1. 3. Ablation of primary IFN release even in the presence of viral growth allows for enhanced virion production. 4. Increased levels of HIV in the microenvironment allows for increased recognition by pDCs. 5. This leads to increased signalling in the pDC even though these cells do not support efficient HIV replication. 6. Increased signalling leads to subsequent release of increased IFN levels. 7. Increased IFN leads to upregulation of TRAIL on pDCs and decrease in bystander CD4+ T cell proliferation and activation.

Our studies have added to the foundation for a general framework for the host- pathogen interaction in regulation of the IFN response to HIV during infection of T cells. First, we show that though little to no IFN is actually produced upon infection of T cells by HIV, host cells are indeed capable of sensing HIV RNA to initiate innate immune responses via IFN induction. We have further shown that the HIV proteins Nef and Vpu can cause IPS-1 degradation, thereby inhibiting RLR-mediated interferon induction. Furthermore, ablation of Vpu and Nef expression in a mutant HIV restores IFN release from T cells during infection. Our current studies are focused on determining if Vpu and Nef degrade IPS-1 using similar mechanisms as in CD4 degradation. Nevertheless, the targeting of IPS-1 by more than one ORF of HIV is a testament to the importance of abrogating this system before IFN begins working to limit the viral infection. Overall, affecting the RLR/IPS-1 pathways will be critical to the spread of virus and the establishment of a pathogenically effective founder population of infected cells in the local microenvironment of infection (Fig 6).

The role of IFN in control of HIV infection and during the development of AIDS has several important facets to consider. Previous studies into HIV pathogenesis have focused on the role of plasmacytoid dendritic cells (pDCs) in response to HIV [40, 41]. In this case, pDCs recognize circulating HIV and/or HIV infected cells leading to an excessive amount of IFN released [42]. Several studies have shown that IFN in this case leads to depletion of the T cell compartments beyond what is caused by just HIV infection. However, the role of IFN in almost every other human pathogen is clear: IFN limits viral infection. So how do we balance this viewpoint with that of our current findings? In addition to pDC cells, almost all cells including other innate immune cells, B and T cells as well as non-immune cells such as fibroblasts can also produce IFN and have the ability to defend themselves against viral infections via IFN signalling. Interestingly, recent studies on other viruses have suggested that IFN produced by virally infected cells through the RIG-I-dependent pathway but not by TLR-dependent pDC cells is essential for host defence against viral infections. These studies have further suggested that the pDC produced IFN may contribute to the development of adaptive immune responses instead of directly controlling local virally infected cells.

The same thing may be true for HIV infection, in which IFN and other innate immune responses by HIV infected cells such as T-cells and macrophages may play more important roles than pDCs in inhibiting HIV replication and prevent the establishment of HIV persistent infection. [43]. These virions are taken up and recognized by plasmacytoid dendritic cells (pDCs) that signal via TLR7 to induce high levels of IFN [40, 41]. This pDC derived IFN is potentially deleterious as it invokes the expression of TRAIL (tumor necrosis factor-related apoptosis-inducing ligand) that can lead to the death of other immune cells including other CD4+ T cells [41, 44]. Enhanced IFN release can also induce high level NK expansion that diminishes over time [45]. These uninfected T cells are also directly slowed down by pDC IFN that lower proliferation and immune potential. Our model does not conflict with the pathologic consequences of pDC activation but rather explains why and how levels of HIV can become so high even when the cells it is infecting should produce IFN to limit viral growth.

It is now possible to begin modelling the host-pathogen signalling network of innate immune response to HIV infection (Fig 6). Positive signals are derived from RIG-I signalling in HIV infected cells that recognizes active, intracellular HIV infection and plasmacytoid dendritic cells via TLR7 signalling recognized infection [44, 46]. However, HIV infection proceeds due to multiple blocks to these signals by HIV working against the innate immune system by Vpu/Nef degradation of IPS-1 and the published observation that Vpr/Vif as well as other mechanisms counteracting IRF3 and NF-κB [36]. IRF-3 has been shown to be destabilized by high dose HIV infection and at least one report has attributed that to Vif or Vpr expression [36, 47]. In addition, others have claimed even RIG-I is modulated by HIV infection even while inducing innate immunity by recognition of HIV genomic RNA [48, 49]. This allows for HIV to replicate unhindered and thereby produce high-level virion release. This is consistent with reports of low IFN released during primary HIV infection however we feel our work using more physiological relevant levels of HIV, with levels of infection between 60 to 100% of infection, may hint at a pathway that is more active even at lower levels of infection [43].

Without induction of IFN, ISGs such as the important antiviral protein APOBEC3G are not potentiated to initiate an anti-HIV state in infected cells allowing HIV to spread unhindered. The dynamics of this network will affect HIV replication, especially establishment of infection during primary HIV infection and ultimately persistence of the HIV infection. Also, this network will impact the replication of other viruses within or in close proximity to HIV infected cells as secondary infections become a major problem for HIV-infected patients. Further studies on the interactions between host innate immune molecules and HIV viral products will fill the gap between primary HIV infection and the well characterized adaptive immune responses. These studies will move to providing insight towards the development of novel approaches against this important human pathogen.
